# Role of Adenoviruses in Cancer Therapy

**DOI:** 10.3389/fonc.2022.772659

**Published:** 2022-06-09

**Authors:** Sintayehu Tsegaye Tseha

**Affiliations:** ^1^ Lecturer of Biomedical Sciences, Department of Biology, College of Natural and Computational Sciences, Arba Minch University, Arba Minch, Ethiopia; ^2^ Department of Microbial, Cellular and Molecular Biology, College of Natural and Computational Sciences, Addis Ababa University, Addis Ababa, Ethiopia

**Keywords:** adenoviruses, cancer therapy, anticancer vaccine, gene therapy, adenoviruse, adenoviral vector

## Abstract

Cancer is one of the leading causes of death in the world, which is the second after heart diseases. Adenoviruses (Ads) have become the promise of new therapeutic strategy for cancer treatment. The objective of this review is to discuss current advances in the applications of adenoviral vectors in cancer therapy. Adenoviral vectors can be engineered in different ways so as to change the tumor microenvironment from cold tumor to hot tumor, including; 1. by modifying Ads to deliver transgenes that codes for tumor suppressor gene (p53) and other proteins whose expression result in cell cycle arrest 2. Ads can also be modified to express tumor specific antigens, cytokines, and other immune-modulatory molecules. The other strategy to use Ads in cancer therapy is to use oncolytic adenoviruses, which directly kills tumor cells. Gendicine and Advexin are replication-defective recombinant human p53 adenoviral vectors that have been shown to be effective against several types of cancer. Gendicine was approved for treatment of squamous cell carcinoma of the head and neck by the Chinese Food and Drug Administration (FDA) agency in 2003 as a first-ever gene therapy product. Oncorine and ONYX-015 are oncolytic adenoviral vectors that have been shown to be effective against some types of cancer. The Chiness FDA agency has also approved Oncorin for the treatment of head and neck cancer. Ads that were engineered to express immune-stimulatory cytokines and other immune-modulatory molecules such as TNF-α, IL-2, BiTE, CD40L, 4-1BBL, GM-CSF, and IFN have shown promising outcome in treatment of cancer. Ads can also improve therapeutic efficacy of immune checkpoint inhibitors and adoptive cell therapy (Chimeric Antigen Receptor T Cells). In addition, different replication-deficient adenoviral vectors (Ad5-CEA, Ad5-PSA, Ad-E6E7, ChAdOx1–MVA and Ad-transduced Dendritic cells) that were tested as anticancer vaccines have been demonstrated to induce strong antitumor immune response. However, the use of adenoviral vectors in gene therapy is limited by several factors such as pre-existing immunity to adenoviral vectors and high immunogenicity of the viruses. Thus, innovative strategies must be continually developed so as to overcome the obstacles of using adenoviral vectors in gene therapy.

## 1 Introduction

In the past few decades, gene therapy for diseases such as cancer using adenoviral vectors has been significantly advanced. Adenoviruses (Ads) can be used either as replication-competent Ads or replication-defective adenoviral vectors for gene therapy (1, 2). The following are the characteristics that made Ads one of the most favorable viruses for gene therapy:-1. Ads have unique ability to infect wide range of cell types (broad cell tropsim) and have the capacity to induce strong cell mediated immunity and humoral response (3); 2. The genetics of Ads has been well known and Ads have stable genome (4); 3. Ads have lytic replication cycle. This lytic replication cycle causes lysis of tumor cells (oncolysis) (5); 4. Adenoviral vectors have low pathogenicity and relatively safe and well-tolerated (4, 6); 5. Ads have large transgene carrying capacity about and can transduce both in dividing and non- dividing cells (4, 7, 8) and 6. Ads can infect professional antigen-presenting cells that are very effective in presenting antigens to T-cells (9). The objective of this review is to discuss current advances in applications of Ads in cancer therapy.

## 2 General Information On Adenoviruses

Ads are non-enveloped viruses with double stranded deoxy ribonucleic acid (DNA) genome. The genome of Ads ranges in size from 26 kb to 45 kb that is encompassed within icosahedral capsid (10, 11). The Ads virion size ranges from 90-100 nm in diameter. There are six kinds of proteins that constitute the adenoviral capsid: penton, fiber, hexon, IX, VIII, and IIIa. The IIIa involves in the assembly of the viral structure. The fiber and penton proteins involve in the attachment and entry of the Ads into host cells and the hexon constitutes most of the viral capsid (12, 13). The proteins IIIa, VIII, and IX make up the virion core, which are associated with the DNA genome. The VIII is important for the stability of the viral capsid (14–16).

The genes of Ads can be categorized into two classes: as early genes (five early genes) and late genes (five late genes). The Ads bind to host cell through its receptors, which includes scavenger receptors, CD46, integrin αvβ5 heparin sulfate proteoglycans, sialic acid etc) and gains entry into the cytoplasm (17–20).

After entry into the target host cell by micropinocytosis (21, 22), Ads first express the five early proteins that are coded by the five early genes (E1A, E1B, E2, E3 and E4 which are involved in protein synthesis and DNA replication. Structural proteins (L1-L5) are coded by the five late genes (15, 23, 24). Following replication, the adenoviral virion leaves the host cell by killing the host cell (lytic cycle). Newly produced Ads can infect wide range of host cells including antigen presenting cells and quiescent cells.

Ads belong to the family adenoviridae that is consisted of five genera: Mastadenoviruses, Avidadenoviruses, Siadenoviruses, Atadenoviruss and Ichtadenoviruse. The genus Mastadenovirus is consisted of Ads that infects humans (human adenoviruses) and non-human primates (25). The genus Aviadenovirus is consisted of Ads that are isolated from birds. Viruses that are isolated from birds, ovine, bovine, deer and possum belong to the genus Atadenoviruses. Ads that are isolated from fish belong to the genus Ichtadenoviruses. The genus Siadenovirus includes viruses that are isolated from invertebrates (25).

Human adenoviruses (HAds) are categorized into seven species (A-G) that are further divided into 57 serotypes (Ad1-Ad57) (1). Adenoviral serotyping is based on capsid proteins (VIII, hexon), phylogenetic distance (≥10%) in adenoviral genes that codes for protease, viral surface antigen neutralizing antibodies, and DNA polymerase (26–28).

HAds have worldwide distribution. Humans can be infected with more than one serotype or species of Ads that are usually acquired in early childhood, which leads to lifelong immunity (29). Ads account for 5% of common cold cases. Wild type Ads often cause mild illness in immunocomptetent individuals, which mainly affects the respiratory tract, eyes and digestive system (30). However, HAds causes severe illness in immunosuppressed individuals (31).

Ads induce diverse innate immune signaling pathways that result in the secretion of a number of proinflammatory cytokines. These proinflammatory cytokines result in the induction of robust adaptive humoral and cellular immune responses. The adaptive immune responses that develop against Ads include both T cells and neutralizing antibodies against the viral surface antigens such as hexon, penton, and fiber proteins (32–35).

Humans can be infected with different kinds of non-human Ads because of their broad tissue tropism and the structural similarity that they have with that of HAds. These characteristics subjected to use the non-human Ads as a vector for gene therapy and recombinant vaccine development so as to overcome the pre-existing antibodies that exists against human adenoviral vectors. There are numerous non-human adenoviral vectors that have used for recombinant vaccine development and gene therapy such as gorilla adenovirus vector (GC-46), chimpanzee adenoviral vectors (ChAdOx1 nCoV-19, ChAd1, ChAd2, ChAd3, ChAd5, ChAd6, ChAd7, and ChAd68); bovine adenoviral vectors; fowl adenoviral vectors; canine adenoviral vectors, ovine adenoviral vectors; porcine adenoviral vectors (36–40).

## 3 Construction Of Adenoviral Vectors

Adenoviruses have been engineered to make them efficient and safe vectors for human use as gene therapy and vaccine vectors by deleting certain genome sequences. So far three generations of adenoviral vectors have been developed to further improve the gene-carrying capacity and safety by deleting more genes. The three generations of adenoviral vectors are as follows:-

### 3.1 First-Generation Adenoviral Vectors

In the first generation, two genes are deleted (E1 and E3) so as to make the adenoviral vector replication defective, but keeping them to transduce host cells without killing them and liberating nearly 8 kb of space in the genome for the transgene and regulatory sequences (41, 42). These adenoviral vectors carry native tissue transduction capability and efficiently express the transgene in target host cells. The major challenge of the first-generation adenoviral vectors is immunogenicity and cellular toxicity.

### 3.2 Second Generation Adenoviral Vectors

In the second-generation adenoviral vectors, in addition to the E1/E3 genes, E2 and E4 regions are also deleted (43, 44). The second-generation adenoviral vectors provide additional space for larger cargo sequences (10.5 kb) and eliminated the possibility of generating replication-competent adenoviruses during amplification. Immunogenicity and cellular toxicity are still a major concern in the second-generation adenoviral vectors (45).

### 3.3 Third Generation Adenoviral Vectors

Third-generation adenoviral vectors are also called high capacity adenoviral vectors (HCAds) because they can accept cargo sequences up to 36 Kb (43, 46–48). The HCAds were generated by deleting all viral sequences except the ITRs and the packaging signal (49). For replication of third-generation adenovirus vectors in cell culture, instead of the complementation by the viral genes encoded by host cells, an additional adenoviral helper virus is provided. Therefore, the third-generation adenoviral vectors are also called helper dependent or gutless adenoviral vectors (50, 51).

Third-generation vectors have several benefits over first and second-generation adenoviral vectors, including less cellular toxicity and reduced immunogenicity and the HCAds can simultaneously encode multiple transgene cassettes (52, 53). The disadvantage of the HCAds is the fact that they are more complicated to first and second-generation adenoviral vectors and also have possibility of helper virus contamination (54).

## 4 Application Of Adenoviruses In Cancer Therapy

### 4.1 Cancer Immune Profiles: Immune Desert, Immune Excluded and Inflamed Tumor

Human cancers can be categorized into three immune profiles (immune phenotypes), depending on their immune status. The three types of cancer immune profiles include: immune deserts tumor, immune-excluded tumors and inflamed tumors. Immune deserts are tumors devoid of immune infiltration, antigen presentation (low major histocompatibility complex-I) and high tumor cells proliferation as seen in tumors such as Head and neck squamous cell carcinomas (HNSCC), glioblastomas, prostate cancer, pediatric malignancies, hormone receptor positive breast cancer (55–57). Immune excluded tumors are tumors with suppressed tumor microenvironment represented by T cells embedded in the tumor stromal microenvironment with high TGF-β signaling, myeloid inflammation, and angiogenesis (58–61). Inflamed tumors have an army of T-cells that are ready to destroy tumors inside the tumor micro-environment. Inflamed tumors are associated primarily with IFN-γ signaling, high tumor PD-L1, TILs, B cells, and intact antigen presentation (i.e., intact HLA and expression of MHC class I on the surface of tumor cells) (58, 62, 63).

The factors that derive suppression of anti-tumor immunity include; I. Increased frequencies of immune suppressive cells (such as T regulatory cells, and Myeloid-derived suppressor cells (MDSCs) (64–66); II. Impairing cytotoxic CD-8+ T-cells (CTLs) activation and infiltration (67–69); III. Up regulating immune checkpoint inhibitors receptors and their ligands (PD-1 and PD-L) (70); IV. Secretion of immunosuppressive cytokines (64, 65); and V. Escaping natural killer cells mediated killing of tumor (71).

Adenoviruses have become the promise of new therapeutic strategy for cancer treatment. Adenoviral vectors can be engineered in different ways so as to change the tumor microenvironment (TME) from cold tumor to hot tumor, including; 1. By modifying Ads to express cytokines, and other immune-modulatory molecules; 2. By modifying Ads to deliver tumor suppressor gene and code for tumor specific antigen. The other way to use Ads in cancer therapy is to use oncolytic adenoviruses, which directly kill tumor cells after replication (72–76)(**Table 1**).

**Table 1:** Clinical trials using replicating and non-replicating adenoviral vectors for cancer therapy.

No	Ad vector	Transgene	Cancer	Phase	Reference
1	ChAdOx1–MVA	STEAP1	Prostate cancer	Preclinical	195
2	Ad-IFN/Syn 3	INF-α	Bladder cancer	Preclinical trail	124
3.	Ad5-yCD/mutTKSR39rep-hIL-12	Cytosine deaminase, HSV-tK, hIL-12	metastatic Prostate cancer	I	NCT03281382
4.	SCH-58500	P53	Primary ovarian cancer, fallopian cancer and peritoneal cancer	I	200
5.	ONCOS-102	GM-CSF	Melanoma	I	127
6.	Adv-tk (GMCI)	Adv-tk	Pediatric brain tumors	I	201
7	Ad-RTS-hIL-12	IL-12	Glioblastoma or malignant glioma; Advanced or metastatic breast cancer; recurrent or progressive melanoma	I	142
8	Ad-E6E7 and MG1-E6E7	HPV E6/E7	HPV-associated cancer	I	194
9	TILT-123	hTNF-α, hIL-2	Advanced melanoma	I	202
10	ADV/RSV-TK	HSV-TK	prostate cancer; glioma, retinoblastoma, mesothelioma	I	156–159
11	BG00001	INF-β	Pleural melanoma	I	
12	DNX-2440	OX40L	Glioblastoma	I	NCT03714334
13	Ad/PNP+ fludarabine	PNP	Head and neck squamous cell carcinoma	1	203
14	ETBX-011, ETBX-061, and ETBX-051 (Tri-Ad vaccine)	TAA	Advanced cancer	I	187
15	Ad5-yCD/mutTKSR39rep-hIL-12	Cytosine deaminase, HSV-tK, IL-12	Prostate cancer	I	NCT02555397
16	Ad5-PSA	PSA	Prostate cancer; recurrent/hormone refractory prostate cancer	I/II	189–191
17	Advexin (rAd-p53)	P53	squamous cell carcinoma of the oral cavity, oropharynx, hypopharynx, and larynx; colorectal cancer, HCC, NSCLC, prostate cancer, breast cancer, ovarian cancer, bladder cancer, glioma, and squamous cell carcinoma of the head and neck	I/II	92–95
18	AdHSV-tk/GCV	HSV-tk Ad- hCMV- Flt3L	High-grade malignant gliomas	I/II	204
19	ETBX-011	CEA	Metastatic colorectal cancer	I/II	183–185
20	Ad-MAGEA3	MAGE-A3	Advanced/Met., MAGE-A3+ Solid Tumors, NSCLC	I/II	NCT02285816
21	LOAd703	CD40L, 4-1BBL	Pancreatic cancer, bacillary cancer, collateral cancer	I/II	NCT03225989
22	Ad-MAGEA3	MAGE-A3	NSCLC	I/II	NCT02879760
23	Adv/tk (GMCI)	HSV-tk	Advanced non-metastatic pancreatic adenocarcinoma II	II	NCT02446093
24	Ad5-SGE REIC/Dk3 (MTG201)	REIC/Dkk3	Relapsed malignant pleural mesothelioma	II	205
25	Adv/HSV-tk	HSV-tk	Metastatic non-small cell lung carcinoma and uveal melanoma	II	NCT02831933
26	DNX-2401		Recurrent glioma	II	NCT02798406
27	Adv/tk	HSV-tk	Advanced hepatocellular carcinoma	III	206
28	rAd-IFN/Syn-3 (instiladrin)	INFα-2b	High grade non-muscle invasive bladder cancer	III	
29	Oncorine or H101		head and neck cancer	III	93, 109
30	Gendicine (rAd-p53)	P53	head and neck squamous cell carcinoma, malignant glioma, HCC, NSCLC and epithelial ovarian carcinoma	III	89–92

### 4.2 Adenoviral Vectors Coding for Tumor Suppressor Protein (p53)

One of the strategies that have been developed to use Ads in cancer therapy is to use replication-deficient adenoviral vectors to carry transgenes that codes for a tumor suppressor protein (p53) or proteins that induce apoptosis or cell cycle arrest (74, 76). Wild-type p53 prevents development of cancer by inhibiting the activation of oncogenes and inducing programmed cell death (apoptosis) when the cell’s DNA repair functions are insufficient to repair DNA damage (77). Suppression of p53 function is common in human cancers and 50% of cancers have mutations in the gene that codes for p53 protein (78, 79). For this reason, the p53 gene has become one of the target genes for transformation research of cancer gene therapy.

Gendicine is a replication-defective recombinant human p53 Ads vector (rAd-p53) expressing p53 proteins which inhibits the uncontrolled division of cancer cells and induces apoptosis of cancerous cells (84). Several studies that investigated the therapeutic efficacy of gendicine against HNSCC have showed good results. Gendicine combined with radiotherapy, chemotherapy, and other conventional treatment regimens demonstrated longer progression-free survival times than conventional treatments alone with no serious side effects except for transient fever or flu-like symptoms (80–87). For example, (80), conducted a clinical trial with 29 patients. Sixteen patients were treated with intratumoral injection of gendicine in combination with radiotherapy and 13 patients were treated with radiotherapy alone. The complete remission rate (also called complete response) in the patients treated with a combination of gendicine and radiotherapy was 5 times higher than the radiotherapy alone group (75% *Vs* 15%) (80).

Gendicine also showed promising results in treatment of cervical cancer patients. The 5-year overall survival rate of gendicine combined with radiotherapy group was 74.2% and the 5-year overall survival rate of the radiation alone group was 56.7%. Both the 5-year overall survival rate and disease free survival rate were significantly higher in the group treated with combination therapy (gendicine combined with radiotherapy) compared to the patients treated with radiation alone (88). In a clinical study of recurrent uterine sarcoma treated with gendicine combined with chemotherapy (89), the remission rate was 66.7%, and the disease control rate was 91.7%.

Gendicine was approved for treatment of squamous cell carcinoma of the head and neck by the Chinese Food and Drug Administration agency in 2003 as a first-ever gene therapy product to be used in combination with chemotherapy and have been in use for more than 15 years (90, 91). Gendicine has been also shown to be effective for treatment of different kinds of cancer in China including malignant glioma, epithelial ovarian carcinoma, Hepatocellular cancer (HCC), and Non-small cell lung cancer (NSCLC) (89–92).

Likewise, Advexin is a replication-defective recombinant human p53 adenoviral vector expressing p53 proteins. Advexin has a deletion on E3 and E1 genes that expresses a functional p53 protein from a Cytomegalovirus promoter (78, 79, 93). Advexin is similar to gendicine except that the p53 in gendicine is expressed from Rous Sarcoma Virus promoter (1). Advexin was proved efficacious against bladder cancer, ovarian cancer, prostate cancer, breast cancer, squamous cell carcinoma of the head and neck, hepatocellular carcinoma, colorectal cancer, squamous cell carcinoma of the oral cavity, oropharynx, hypopharynx, and larynx and non-small cell lung cancer (NSCLC) (92–95).

### 4.3 Oncolytic Adenoviruses as Anticancer Virotherapy

Oncolytic viruses are viruses that that specifically infect and replicate in a tumor cells and kill the cancer cells by their lytic replication (73, 75, 96). Oncolytic Ads, particularly oncolytic HAds are one of the leading candidate viruses for cancer virotherapy because of their good safety profile and high immunogenicity. Oncolytic Ads are genetically engineered Ads which acquired traits that enables them to infect and preferentially replicate in tumor cells (97). Oncolytic adenoviral vector technologies have been approved in some countries for treatment of cancer in humans (1, 75, 98). As compared with normal and quiescent cells, generally, tumor cells are more permissive to Ads (99), because of different reasons. The first reason is that the entire pattern of gene expression in cancer cells is conducive for Ad replication (100). The second reason is the fact that specific viral entry receptor is highly expressed in tumor cells. The other reason is the higher cell division and metabolic rate that take place in cancerous cells than that of normal and quiescent cells (101, 102). The advantage of an oncolytic Ads is not only to specifically replicate in and lyse tumor cells, but oncolytic adenoviruses can also stimulate potent anti-viral and anti-tumor immune responses for tumor-specific antigens that are released following lysis of Ads infected tumor cells (103–105).

Adenoviral vectors have been engineered to efficiently undergo oncolytic replication in cancer cells without replicating in healthy cells (106, 107). For example, ONYX-015 with a partial E1B gene deficiency is oncolytic adenoviral vector that infects and replicate in tumor cells that lacks p53 but unable to replicate in healthy cells expressing p53 (106). ONYX-015 has been demonstrated to be effective and well-tolerated oncolytic adenoviral vector that is reported to be more effective when given in combination with different cancer chemotherapies (107, 108). Oncorine (H101) is also a genetically modified oncolytic adenoviral vector expressing p53 gene. The Chinese food and drug administration agency has approved Oncorin for the treatment of head and neck cancer in combination with chemotherapy (93, 109).

### 4.4 Adenoviruses Expressing Immunomodulatory Molecules

Adenoviruses can be used in cancer therapy by modifying the viruses to stimulate antitumor immune response in different ways including by expressing cytokines, and other immune-modulatory molecules (72, 110–112).

#### 4.4.1 Interferon Armed Adenoviruses

Interferons (IFNs) are the first group of cytokines that demonstrated efficacy in the treatment of malignancies. IFN signaling is mediated by binding of IFNs to their receptors and subsequent activation of Janus tyrosine kinase (JAK)-STAT signaling pathway (113). Interferon (IFN) has a strong antitumor effect and has been used in the treatment of pancreatic cancer. Some studies showed that IFN-α significantly prolongs survival rate (by 2 to 5 years) (114–116). However, there are limitations in using IFN-based therapies, including dose-limiting systemic toxicities and low intratumoral concentration of IFN because of its short half-life in the bloodstream (117, 118). In response to this, oncolytic adenoviruses have been engineered to express IFN, which showed positive outcomes in treatment of cancer.

Armstrong et al. (119, 120) has reported oncolytic adenoviruses expressing human IFN-α as a promising platform for selective, long-term expression of IFN in human pancreatic cancer tissues (119, 120). Armstrong et al. used the oncloytic adenovirus Ad5/Ad3-Cox2-ΔE3-ADP-IFN in their study, which was developed to selectively replicate within cancer cells expressing cyclooxygenase 2 (Cox2). In order to improve the infectivity and oncolysis of the oncolytic adenoviruses, they made genetic modification in the virus to include an Ad5/Ad3 chimeric fiber and overexpress the adenovirus death protein (ADP). The expression of adenovirus death protein (ADP) occurs during the late stage of infection a lytic infection. ADP promotes the release of progeny virus (virion) by accelerating the lysis and death of the host cell **(** 121).

Similarly, other researchers have also reported as oncolytic adenoviruses expressing IFN-α have promising outcomes in treatment of cancer. The researchers reported that an oncolytic adenovirus (OAd-hamIFN) that was investigated in an immunocompetent Syrian hamster model of pancreatic ductal adenocarcinoma showed efficient viral replication in tumor, significant inhibition of tumor growth, and enhanced survival when used in combination with chemo therapies and radiation therapies (122, 123). Likewise, studies conducted by Tao etal. (124) have shown that significant tumor regression of bladder cancers occurred following administration of an adenovirus expressing human interferon α (Ad-IFNα) using a mouse superficial bladder cancer model in which human bladder tumors are growing (124).

#### 4.4.2 GM-CSF Expressing Adenoviruses (ONCOS-102)

Granulocyte macrophage colony-stimulating factor (GM-CSF) promotes activation of T-cells and maturation of dendritic cells (125, 126). Therefore, an immunotherapy using adenoviruses that express GM-CSF transgene can be an effective anti-tumor therapy. ONCOS-102 is an oncolytic adenovirus that contains GM-CSF transgene (127). A clinical study (phase-I) in patients with advanced solid tumors including colon, lung, and ovarian cancers demonstrated a strong immune cell infiltrate into tumors without dose-limiting toxicities (127).

#### 4.4.3 LOAd703 Expressing 4-1BBL and Trimerized CD40L

The other oncolytic adenovirus that expresses immunostimulatoy cytokine is LOAd703. LOAd703 is armed with 4-1BBL and trimerized CD40L that was shown to replicate and kill pancreatic cancer cells *via* oncolysis in both *in vitro* and *in vivo* assays (128).

#### 4.4.4 TILT-123 (Expressing TNF- α)

TILT-123 is an oncolytic adenovirus that incorporates transgenes for human tumor necrosis factor alpha (TNF-α) and interleukin-2 (IL-2). TNF-α and IL-2 were shown to be promising T cell stimulating factors when used in combination with adoptive cell therapy (129, 130).

#### 4.4.5 IL-12 and Other Cytokines Armed Adenoviruses

Interleukin-12 (IL-12) is a proinflammatory cytokine that initiates antitumor immune responses by promoting the generation of tumor-specific cytotoxic T lymphocytes (CTLs) and activating natural killer (NK) cells and CD4+ T cells (131, 132). Several studies demonstrated promising antitumor effects of IL-12 in mice having solid tumor and hematologic malignancies (133–137). However, findings from clinical trials indicated severe side effects of systemic administration of IL-12 that markedly dampened hopes of the successful use of this cytokine in cancer patients (138). But, the use of Ads containing IL-12 transgene seems critical for maximizing the density of IL-12 that reaches the tumors and alleviating the toxicity.

Ads that were engineered to express IL-12 have been shown to enhance immune stimulation and antitumor effect in a clinical trial (139) and pre-clinical studies (140). Likewise, a study by Wang et al. showed that oncolytic adenovirus (Ad-TD-nsIL-12) expressing IL-12 induces strong antitumor immune response against pancreatic cancer in Syrian hamster models without toxicity (141). Similarly, a replication-deficient adenoviral vector encoding human IL-12 p70 transgene (Ad-RTS-hIL-12) was also shown to have no toxic effect in phase one clinical trial (142). A recent trial found that intratumoral injection of Ad-RTS-hIL-12 was safe in patients with recurrent glioblastoma (142).

In addition to IL-12, there are also other cytokines, such as IL-24 and IL-13 that have been used to arm adenoviruses and have shown promising immune-activating properties in multiple preclinical cancer models (143–145). RANTES is another cytokine engineered in Ad that has been shown to enhance anticancer effect. In murine models of mammary adenocarcinoma and lymphoma, Ad-RANTES-E1A eradicated established tumors and inhibited metastases by recruiting DCs, macrophages, NK cells, and CD8+ T cells into the immunologically cold tumors (146).

#### 4.4.6 Adenoviruses Armed With Bispecific T Cell Engager (BiTE)

Bispecific T cell engager (BiTE) is a kind of artificial antibody that represents an innovative immunotherapy approach which enhances patients’ immune response to tumors. BiTE has dual antigen specificity, allowing them to bind to two unique antigens at the same time, i.e. the BiTE bind simultaneously to both tumor associated antigen and T cell (usually CD3), ultimately stimulating T-cell activation, tumor killing and cytokine production (147). BiTE has been shown to be promising immunotherapy for the treatment of cancer in preclinical and clinical studies (148, 149). The therapeutic efficacy of BiTE can be improved by using BiTE in conjunction with adenoviruses.

Pomés et al. tested weather an OAd expressing FAP-targeting BiTE improve antitumor efficacy (150). FAP-BiTE (FBiTE) comprises two single chain variable fragments (ScFvs) joined by a flexible Gly-Ser linker. One scFv arm binds mouse and human Fibroblast Activation Protein (m/hFAP), while the other binds human CD3epsilon on the T cell receptor (TCR). The study showed that the FBiTEs activate and re-direct T-cells to FAP+ cells, which leads to enhance cytotoxicity *in vitro* and improve antitumor efficacy *in vivo*.

Likewise, Alemany group engineered an oncolytic adenovirus expressing an EGFR-targeting BiTE that showed improved T cell-mediated killing of cancer cells both *in vivo* and *in vitro* (151). They also demonstrated that anti-EGFR BiTE-armed OAd in combination with adoptive CAR-T cell therapy results in improved antitumor efficacy and prolonged survival of mice as a result of intratumoral T cell activation by BiTE (152).

Similarly, another research group (Fisher group) that developed BiTE armed oncolytic adenovirus (EnAd-SA-EpCAM) reported promising results in use of BiTE expressing adenoviruses against cancer (153). The BiTE of EnAd-SA-EpCAM binds to epithelial cell adhesion molecule (EpCAM) in cancer cells. The Fisher group reported that the EnAd-SA-EpCAM effectively activate endogenous T cells within the immune-suppressive microenvironment and exhibited killing of endogenous tumor cells without the addition of exogenous T cells (153).

Recently, the Fisher group engineered another BiTE armed oncolytic adenovirus (EnAd-FAP-BiTE), which is targeted fibroblast activation protein (FAP) in cancer-associated fibroblasts (CAFs). CAFs are the main cellular component of solid tumor TME. The EnAd-FAP-BiTE induced the activation of tumor-infiltrating T cells that target and kill CAFs (154). Likewise, another BiTE armed adenovirus (ICO15K-FBiTE) that was developed by the Fisher research group was shown to enhance overall antitumor efficacy without increasing the toxicity in mouse model (154).

#### 4.4.7 ADV/HSV-TK

ADV/HSV-TK is an adenoviral vector expressing the herpes simplex virus (HSV) thymidine kinase (TK) gene. The HSV-TK protein has two principal functions, including 1. TK is a superantigen that stimulates a potent immune reaction and 2. A nucleotide analog product of prodrug phosphorylation lead to the death of dividing cancer cells (155). Herman et al. studied ADV/HSV-TK in combination with ganciclovir for the treatment of human prostate cancer (156). They reported that injection of ADV/HSV-TK into the prostate gland in the region with the greatest concentration of tumor cells resulted in significant reduction in tumor burden. Herman et al. also showed that ADV/HSV-TK was proven safe, with minimal toxicity (156). Likewise, other researchers also made similar observation on the efficacy and toxicology of ADV/HSV-TK against glioma, retinoblastoma, and mesothelioma (157–159).

### 4.5 Combination Therapy Using Adenoviruses and Chimeric Antigen Receptor (CAR) T Cells

One of the strategies that have been used for the immunotherapy of cancer is adoptive cell therapy, that includes chimeric antigen receptor (CAR) T cells, tumor-infiltrating lymphocytes (TIL), and T cell receptor modified (TCR) T cells (160). TCR-T cells are designed to encode receptors that specifically recognize cancer-specific antigens, and function through Major Histocompatibility Complex (MHC)-dependent mechanism, that limits their use (161). Whereas, CAR-T cell therapy functions through MHC-independent mechanism. CAR-T cell has been effective in the treatment of different types of cancer, which includes chronic lymphocytic leukemia and non-Hodgkin’s lymphoma (162, 163). However, the use of CAR-T cells as a monotherapy has not demonstrated much success in solid tumors because of immunosuppressive tumor microenvironment (TME) and poor tumor infiltration of CAR-T cells (164). Combination therapy with adenoviruses viruses provides one potential strategy for improvement of CAR-T therapy in solid tumors.

A study conducted by Watanabe and his colleagues demonstrated that the combination of oncolytic adenoviruses (expressing TNF-α and/or IL-2) and mesothelin-redirected CAR-T cells (meso-CAR-T) overcomes the immunosuppressive nature of the pancreatic cancer TME (165). Watanabe et al. demonstrated that tumors treated with the combination of the virus expressing TNF-α and IL-2 (Ad5/3-E2F-d24-TNF-α-IRES-IL-2 (OAd-TNFα-IL2) and meso-CAR-T cells were infiltrated with significantly more CD4+ and CD8+ T cells compared to monotherapy with meso-CAR-T cells, or a combination with meso-CAR-T cells and the parent adenovirus lacking cytokine expression (165). They also showed that meso-CAR-T cells in combination with OAd-TNFα-IL2 resulted in significantly higher accumulation of CAR-T cells at the tumor site when compared to meso-CAR-T monotherapy.

Likewise, a study that was done by a Suzuki group demonstrated better efficacy of CAR-T cell in treatment of prostate cancer when used in combination with oncolytic adenoviruses. In order to improve the efficacy of the CAR-T cell therapy, this research group (Suzuki group) used a combinatorial adenovirus vector (oncolytic adenovirus (Ad5Δ24) and helper-dependent adenovirus expressing a mini anti-PD-L1 antibody (HDAdPD-L1) collectively termed CAd-VECPDL1) in combination with human epidermal growth factor receptor 2 (HER2)-specific CAR-T cells (166). The findings showed that using this combination in an NSG mouse model was more effective in reducing tumor size and prolong survival of mice with prostate cancer (166).

The Suzuki group further modified the CAd-VECPDL1 vector by incorporating IL-12 (CAdVECIL12_PDL1) and tested it in a head and neck squamous cell carcinoma (HNSCC) model (167, 168). In a xenograft model of NSG mice (NOD scid gamma mouse), the combination of HER2-CAR-T cells and CAdVECIL12_PDL1 virus significantly prolonged survival of treated animals to more than 100 days as compared to 21–24 days in the control groups, and HER2-CAR-T cells were detected in the tumors of surviving mice over 100 days after initial therapy (168). The research group also used an orthotopic HNSCC model, establishing both primary tumors and lymphatic metastases, to test the aforementioned combination therapy. Mice that received both HER2-CAR-T cells and CAdVECIL12_PDL1 had improved tumor growth control at both primary and metastatic sites, maintained body weight, and had prolonged survival when compared to untreated and monotherapy groups (168). Taken together, combination therapy with oncolytic viruses provides one potential strategy for improvement of CAR-T therapy in solid tumors.

Tumor xenograft animal models provide us a research tool for preclinical drug response evaluation by determining anti-tumor efficacies; toxicity, tumorgenesis, pharmacokinetics and pharmacodynamics (169, 170). In addition, this research tool enables to have a better understanding on the involvement of certain oncogenes or tumor suppressors in tumor development (170). Mice are the most commonly used animals for tumor xenograft models. The advantageous of using mice as tumor xenograft models include: - I. The presence of comparable genome size with humans, II. Short reproductive cycle, III. Large litter size; IV. Low cost; V. Ease of manipulation (170).

### 4.6 Combination Therapy Using Adenoviruses and Antibodies Against Immune Checkpoint Proteins

#### 4.6.1 Immune Checkpoint Inhibition

Immune checkpoints are regulators of the immune system. Immune checkpoints pathways prevent the immune system from attacking its own cells. Immune checkpoints works by means of immune check point proteins, including programmed cell death 1 protein (PD-1) and programmed cell death protein-ligand 1 (PD-L1), and cytotoxic T-lympotcyte antigen-4 (CTLA-4). One of the mechanisms by which tumor cells evade immunesurveillance is by activation of immune checkpoint pathways that suppress antitumor immune responses. PD-1 is the transmembrane programmed cell death 1 protein, which interacts with PD-L1 (PD-1 ligand 1, or CD274). The binding of PD-L1 on cancerous cell with PD-1 on T-cell surface results in inhibition of T-cell activity. Therefore, antibodies that bind with PD-L1 and or PD-1 can block the interaction of PD-L1 and PD-1, allowing T-cells to attack the tumor.

Immune checkpoint inhibitors therapy is a kind of immunotherapy that work by blocking the binding of checkpoint proteins (PD-1, PD-L and CTLA-4) with partner proteins so that T-cell became free and active to attack cancer cells. Ipilimumab is an anti-CTLA-4 antibody (that blocks CTLA-4 ligand and prevents inhibition T-cells) that was approved by the United States food and drug administrations (U.S. FDA) in 2010 for the treatment of advanced melanoma (171). However, the systemic administrations of immune checkpoint inhibitors have been shown to cause severe immune-related adverse events (172–174). One of the strategies that can be employed to overcome these obstacles of using immune checkpoint inhibitors (such as anti-PD-L1, anti-PD-1 and anti-CTLA-4) in cancer therapy is to utilize immune checkpoint inhibitors in combination with oncolytic adenoviruses.

##### 4.6.1.1 Ad5/3-Δ24: Adenovirus Expressing Anti-CTLA-4 Antibodies

Ad5/3-Δ24 is an oncolytic adenovirus that was engineered to code for anti-CTLA4 antibody (175). Promising results have been achieved with the oncolytic adenovirus armed with anti-CTLA-4 antibodies (Ad5/3-Δ24) in mouse model. The local expression of anti-CTLA-4 antibody following administration of Ad5/3-Δ24-CTLA4 resulted in activation of T cells (175). In addition, a significantly higher concentration of antitumor antibody was produced, while plasma levels remained at safe concentrations. The anti-CTLA-4 antibodies also showed direct proapoptic effect both *in vivo* and *in vitro* (175).

##### 4.6.1.2 Ad5-CMV-mIL2 and Ad5-CMV-mTNF- α in Combination With Anti-PD-1 Antibodies

Cervera-Carrascon and his colleagues that tested nonreplicating vectors expressing IL-2 (Ad5-CMV-mIL2) and TNF-α (Ad5-CMV-mTNF- α) in combination with programmed cell-death protein 1 (PD-1) blocking antibodies in a mouse model demonstrated complete regression of murine melanoma tumors, and prolonged survival of mice (176). Furthermore, they showed that the viral infection shifted the cytokine profile of the tumor microenvironment towards T-helper type 1, indicating that non-replicating adenoviral vectors significantly improve antitumor immunity (176).

##### 4.6.1.3 DNX-2401 (Tasadenoturev) in Combination With Pembrolizumab

DNX-2401 is a replication-competent oncolytic adenovirus that selectively infects cancer cells lacking the normal retinoblastoma (Rb) protein signaling pathway (177). Aiken et al. tested DNX-2401 in combination with intravenous pembrolizumab (PD-1 immune checkpoint inhibitor) in patients with recurrent glioma and the findings showed that treatment of glioma with combination of DNX-2401 and pembrolizumab significantly improves the disease burden (178).

##### 4.6.1.4 ONCOS102 in Combination With Pembrolizumab

Li et al. investigated the recombinant oncolytic adenovirus ONCOS-102 in combination with the antibody pembrolizumab (PD-1 immune checkpoint inhibitor) in patients with locally advanced or unresectable melanoma (161). They reported that combination therapy with ONCOS-102 and pembrolizumab resulted in regression of the disease and increases in circulating proinflammatory cytokines, and tumor specific T cells without dose limiting toxicities (161).

### 4.7 Adenoviral Vectors as Recombinant Anticancer Vaccines

Adenoviral vectors can also be used as a platform for anticancer vaccine development. This is based on the fact that adenoviral vectors can be engineered to stimulate antitumor immune response by expressing tumor-antigens. Replication-deficient adenoviral vectors are one of the viral vectors that have been extensively used as recombinant cancer vaccines as they cause potent cell mediated (cytotoxic CD8 T cells responses) and humoral immune response against transgenes expressed by the adenoviral vectors (1).

Cytotoxic CD8+ T cells are major constituent of anti-cancer immunity. The TCR of CD8+ T cells recognize tumor antigen presented by MHC-I and when bound, the cytotoxic CD8+ T cells triggers its cytotoxic activity. However, some tumor cells lower their MHC-I expression and avoid being detected by cytotoxic CD8+ T cells (179, 180). Another way that tumor cells use to escape cytotoxic CD8+ T cells by is to stop expressing molecules essential for co-stimulation of cytotoxic CD8+ T cells such as CD 86 or CD80 (181, 182).

#### 4.7.1 ETBX-011(Ad5-CEA)

ETBX-011(also called Ad5-CEA) is an adenoviral vector-based cancer vaccine that is engineered to express a modified carcinoembryonic antigen (CEA) which contains the highly immunogenic epitope CAP1-6D. CEA is found in different kinds of cancer cells. ETBX-011 induces potent CEA-specific cell-mediated immune responses with antitumor activity (183). ETBX-011 is well-tolerated in metastatic colorectal cancer patients and has potential survival benefit (184, 185). This study showed evidence of a potential survival benefit: 25 patients treated at least twice with ETBX-011 exhibited a 12-month overall survival probability of 48% and a mean overall survival of 11 months (184, 185).

In order to overcome the challenge posed by tumor heterogeneity, such as the diversity of tumor associated antigens (TAA), a Tri-Ad vaccine (a combination of ETBX-011 with three different human TAA-expressing Ad vector vaccines (ETBX-011, ETBX-061, and ETBX-051) have been developed and tested in phase I clinical trial. The first, ETBX-061, is an Ad5-based adenovirus vector vaccine with the same backbone as the ETBX-011, but expressing a modified human mucin 1 (MUC1) gene. The modified MUC1 gene contains agonist epitopes designed to increase CTL antitumor immune responses. The other, ETBX-051 or Ad5-brachyury, encodes the entire brachyury gene with a deletion of 25 amino acids involved in DNA binding, and modified to express an enhancer T cell epitope (186). The Tri-Ad vaccine regimen induces antitumor cytotoxic T cell (CTL) responses and was proven safe and well tolerated in treatment of advanced cancer (187).

#### 4.7.2 Ad5-PSA

Ad5-PSA is replication-deficient adenoviral vector that is engineered to express human prostate specific antigen (PSA). Ad5-PSA stimulates potent anti-PSA T cell responses and causes the destruction of PSA-secreting tumor cells both in preclinical (188) and clinical (189–191). Furthermore, Ad5-PSA prolongs survival and was demonstrated safe in patients with recurrent and hormone refractory prostate cancer (189–191).

#### 4.7.3 Ad-E6E7 in Combination With and Ad-MAGEA3

Ad-E6E7 is another replication-deficient adenovirus-based anti-cancer vaccine, which expresses human papillomavirus (HPV) genes E6 and E7 (192, 193). MG1-E6E7 is an oncolytic maraba virus strain which also expresses the HPV genes E6 and E7. The combination of Ad-E6E7 and MG1-E6E7 induced potent tumor-specific responses in various mouse cancer models (194). In addition, the combination of Ad-E6E7 with MG1-E6E7 was proven to significantly prolong survival of mice with HPV-associated cancer (194).

#### 4.7.4 ChAdOx1–MVA

Cappuccini et al. investigated the immunogenicity and efficacy of ChAdOx1–MVA against prostate cancer in mouse model (195). They reported that the ChAdOx1–MVA induced tumor specific cell mediated immune response. Furthermore, the ChAdOx1–MVA was proven to prolong the survival of the mice when used in combination with anti-PD-1 antibody (195).

#### 4.7.5 Adenovirus-Transduced Dendritic Cells

The other approach to use adenoviral vectors for anticancer vaccine is the use Dendritic cells (DC)-based adenoviral vaccines. In this approach, DCs are transduced *ex vivo* with Ads encoding cancer specific antigens. The advantageous of transducing DC ex vivo instead of injecting Ads *in vivo* include: 1. *Ex vivo* transduction of DC overcomes pre-existing anti-viral immunity and induce effective anti-tumor responses (196, 197) and 2. *Ex vivo* transduced DC induces lower anti-viral antibody responses than that of Ads injected *in vivo* (196, 198).

Ad-transduced DCs have shown positive outcomes against both solid tumor and hematologic cancers (4, 66, 199). Butterfield et al. (199) tested an autologous DCs transduced *ex vivo* with Ads encoding the full-length melanoma antigen MART-1/Melan-A. They reported that the autologous DC-based adenoviral vaccine significantly induced cell mediated immune response (CD+8-T cells response) in metastatic melanoma patients (199). Similarly, in patients with advanced NSCLC, injections of autologous DCs resulted in induction of systemic tumor antigen-specific immune responses with enhanced CD8+T-cell infiltration (4).

#### 4.7.6 Recombinant Gorilla Adenovirus HPV Vaccine (PRGN-2009)

Pellom et al. evaluated PRGN-2009, a therapeutic gorilla adenovirus HPV vaccine containing multiple cytotoxic T cell epitopes of the viral oncoproteins HPV 16/18 E6 and E7, including T cell enhancer agonist epitopes. The study revealed that PRGN-2009 treatment reduced tumor volume and increased CD8+ and CD4+ T cells in the tumor microenvironment of humanized mice bearing the human cervical tumor SiHa. The PRGN-2009 monotherapy in the syngeneic TC-1 model also reduced tumor volumes and weights, generated high levels of HPV16 E6–specific T cells, and increased multifunctional CD8+ and CD4+ T cells in the tumor microenvironment (40).

## 5 CHALLENGES AND SOLUTIONS OF USING ADENOVIRAL VECTORS IN CANCER THERAPY

The application of adenoviral vectors in cancer therapy and vaccine development is severely hampered by different factors including pre-existing immunity to the most common Ad vectors infecting the human population (207, 208); immunodominance of adenoviral antigens over the vaccine transgene antigen(s), and heterologous immunity with other pathogens (209, 210). The following are the details of the challenges and solutions of using adenoviral vectors in cancer therapy and vaccine development.

### 5.1 Pre-Existing Immunity in the Host

Pre-existing immunity of Ad vectors infecting the human population is the major factor that hampers the application of adenoviral vectors in cancer therapy and vaccine development (207, 208). The HAdV5 is the common serotype that infects humans, especially in developing countries (33, 211). HAd capsid proteins, particularly the hexon protein is very immunogenic (212). The host’s adaptive immunity arm detects the hexon protein and releases serotype-specific neutralizing antibodies (nAb) that block a post-entry step (213, 214). Therefore, during second contact with the same adenovirus serotype, the host nAb could rapidly neutralize it. In addition to the humoral immune response (antibodies), strong and sustained CD8+ T-cell responses follow adenoviral infections (3). Up to one-third of circulating T-cells against HAd have been reported to be CD4+ T-cells specific for a hexon epitope conserved between HAd serotypes. Thus, the host’s preexisting CD4+ T-lymphocytes might promptly respond to various subsequent adenovirus serotypes in either blood or gut (215). The following are the strategies that have been used to overcome the challenges of preexisting immunity in using adenoviral vectors.

#### 5.1.1 Use of Non-Human Adenoviral Vectors

Non-human adenoviruses have been used to overcome the challenge of preexisting human adenoviral immunity. The commonly used nonhuman adenovirus vectors include gorilla adenovirus and chimpanzee-derived adenovirus vector (ChAd) (40). Several chimpanzee adenoviral vector-based vaccines, such as ChAd7 for Ebola virus, ChAd6 for rabies, and ChAd6, ChAd7, and ChAd9 for malaria, have shown high efficacy in animal models (216). Furthermore, ChAd63-based malaria and ChAd3-based hepatitis C virus vaccines have shown to be safe and highly immunogenic in phase I clinical trial (217, 218). Despite low seroprevalence of ChAd vectors in humans, pre-existing cross-reactive T cells against many conserved viral antigens are still a major concern. HAd-induced ChAd cross-reactive T cells have been reported against ChAd6, ChAd7, ChAd24, ChAd32, and ChAd68 (35, 38, 219, 220).

In addition to chimpanzee adenoviruses, several other adenoviruses derived from animals such as bovine, porcine, ovine, canine, and fowl are have been explored for vector development (221–224). The human population lack nAbs against these adenoviruses, and therefore, the vectors derived from these adenoviruses could be more efficacious in comparison to HAd and ChAd. The mouse models with experimentally induced pre-existing immunity by the administration of HAd vector demonstrated a lack of nAbs and CD4+ T cells against PAd3 and BAd3 (225). Furthermore, BAd3- or PAd3-based influenza virus vaccine demonstrated high efficacy even in the presence of pre-existing HAd5 immunity. There was also no effect of pre-existing HAd5 immunity on transgene expression, immunogenicity, and efficacy in animal models.

#### 5.1.2 Heterologous Prime-Boost Strategy

Heterologous prime-boost strategy is another strategy to avoid pre-existing adenoviral vector immunity. Unlike homologous prime-boost immunizations, in this strategy, the priming and boosting are done by using different antigen delivery vehicle and/or vectors derived from either different serotypes of the same species or vectors from completely different host species. For instance priming with ChAd68 and boosting with ChAd1 or priming with DNA and boosting with HAd5 (226). Studies have shown that the heterologous prime-boost induces more robust immune responses compared to single vaccination or homologous prime-boost immunizations. The cellular immune responses induced by DNA prime and HAd boost were not affected by pre-existing HAd5 immunity. A preclinical study involving Plasmodium or SARS antigens encoded by Modified Vaccinia Ankara (MVA)/adenoviral vector as prime/boost showed induction of robust T cell and Ab responses of higher magnitude compared to Ad/DNA regimens (218, 227–229).

#### 5.1.3 Routes of Immunization

Studies showed that different route of vaccinations can overcome the detrimental effect of pre-existing immunity. This result is partly due to evasion of tissue-resident Ad-specific T cells when using different routes of immunization. Tissue resident CD8 memory T cells are not systemic and do not prevent Ad vector infection in distant tissues (230–232). In preclinical study, HAd5-induced protective immune responses by intranasal/intratracheal immunization were not affected by pre-existing HAd5 immunity that had been induced by intramuscular administration of an unrelated HAd5 vector (233).

### 5.2 Immunodominance Over Transgene Immunity

Adenoviruses induce potent cell and antibody immune responses. A study has shown that adenoviral-derived epitopes can dominate over the transgene-derived epitopes and hinder the induction of transgene-specific immunity. This impairment of transgene-specific immune responses in naive vaccines is due to immune competition. Epitopes derived from an adenovirus vector were shown to inhibit the induction of HIV GagL85-93-specific CD8+ T cells (234). This study demonstrated that competition occurs at the level of responding CD8+ T cells, and co-immunization with an interleukin 2-encoding plasmid restored GagL85-93-specific CD8+ T cell responses in the presence of an adenoviral hexon486-494 epitope. The study suggests that adenoviral antigen-specific T-cell immunity is primed efficiently during adenoviral vector-based immunization, which can limit the immunogenicity of adenoviral vector-encoded transgenic antigens. This study suggest the need for modifications of adenoviral vector or the transgene used in immunization to dominate over the adenoviral vector-specific epitopes and induce more effective transgene specific immunity.

### 5.3 Heterologous Immunity Induced by Adenoviral Antigens

The other challenge of using adenoviral vectors in gene therapy is cross-reactive immune response induced by adenoviruses. For example, nAbs and T cells against HAd2 cross react with HAd35 and reduce the immunogenicity and efficacy of the HAd35 vectors (235, 236). Other studies showed that nAbs and T cells against HAd cross react with ChAd (ChAd6, ChAd7, ChAd24, ChAd32, and ChAd68) (35, 38, 219, 220). Adenoviruses can also induce robust cross-reactive immune responses against unrelated pathogen such as hepatitis C virus (HCV) antigens (209). Therefore, a careful evaluation of adenoviral-induced cross-reactive immune responses is needed before using adenoviruses in gene therapy and vaccine development.

## 6 Conclusions

Adenoviruses viruses have become the promise of new therapeutic strategy for cancer treatment. Adenoviral vectors can be engineered in different ways for cancer treatment including; 1. By modifying adenoviruses (Ads) to deliver transgenes that codes for tumor suppressor gene (p53) and other proteins whose expression result in cell cycle arrest; 2. By modifying Ads to express tumor specific antigens, cytokines, and other immune-modulatory molecules; 3. The other strategy is using oncolytic adenoviruses. However, the use of adenoviral vectors in cancer therapy is limited by several factors such as pre-existing immunity to adenoviral vectors; immunodominance of adenoviral antigens over the vaccine transgene antigen(s); and heterologous immunity with other pathogens. Thus, innovative strategies must be continually developed so as to overcome the obstacles of using adenoviral vectors in gene therapy.

## Author Contributions

The author confirms being the sole contributor of this work and has approved it for publication.

## Conflict of Interest

The authors declares that the research was conducted in the absence of any commercial or financial relationships that could be construed as a potential conflict of interest.

## Publisher’s Note

All claims expressed in this article are solely those of the authors and do not necessarily represent those of their affiliated organizations, or those of the publisher, the editors and the reviewers. Any product that may be evaluated in this article, or claim that may be made by its manufacturer, is not guaranteed or endorsed by the publisher.
